# Effect of Fluorosilicone Rubber on Mechanical Properties, Dielectric Breakdown Strength and Hydrophobicity of Methyl Vinyl Silicone Rubber

**DOI:** 10.3390/polym15163448

**Published:** 2023-08-18

**Authors:** Zhaoyang Wang, Yankai Lin, Zhanxu Li, Yumeng Yang, Jun Lin, Shaojian He

**Affiliations:** 1 State Key Laboratory of Alternate Electrical Power System with Renewable Energy Sources, North China Electric Power University, Beijing 102206, China; wang94269264@163.com (Z.W.); zhanxuli0614@163.com (Z.L.); yumengyang84@163.com (Y.Y.); 2 Jiangmen Power Dispatching Center of Jiangmen Power Grid, Jiangmen 529000, China; kyoexii@sina.com

**Keywords:** silicone rubber, fluorosilicone rubber, mechanical properties, breakdown strength, hydrophobicity

## Abstract

Silicone rubber (SIR) is used in high-voltage insulators because of its insulation, and excellent hydrophobicity is very important in harsh outdoor environments. To enhance the hydrophobicity and low-temperature resistance of silicone rubber, methyl vinyl silicone rubber and fluorosilicone rubber (FSIR) blend composites with different ratios were prepared. The samples were characterized and analyzed using scanning electron microscopy, tensile testing, dynamic mechanical analysis and static contact angle testing. The results showed that after blending, SIR and FSIR were well compatible. FSIR had higher elastic modulus and reduced the tensile strength to some extent in SIR/FSIR composites. The addition of a small amount of FSIR made its crystallization temperature decrease from −30 to −45 °C, meaning that the low-temperature resistance was significantly improved. The breakdown strength of SIR/FSIR composites can still be maintained at a high level when a small amount of FSIR is added. The contact angle of the composites increased from 108.9 to 115.8° with the increase in FSIR content, indicating the enhanced hydrophobicity. When the samples were immersed in water for 96 h, the hydrophobicity migration phenomenon occurred. The static contact angle of the samples with less FSIR content had a weaker decreasing trend, which illustrated that the hydrophobicity was maintained at a high level.

## 1. Introduction

Hydrophobicity materials are widely used in all aspects of industry and life, such as high-voltage insulators, hull surface coatings, oil pipeline inner walls and so on. An insulator is an important and indispensable part of power transmission and transformation lines, and the operation condition is directly related to the stability and safety of the power grid [1,2]. The silicone rubber composite insulator is the latest insulator. Compared to the traditional porcelain insulator and glass insulator, the silicone rubber composite insulator has the advantages of light weight, favorable durability, excellent hydrophobicity, good resistance to dirt flash and being easy to manufacture and maintain [3,4,5,6,7,8]. Silicone rubber composite insulators are mainly composed of high-temperature vulcanized silicone rubber (HTV) composite umbrella skirt, glass fiber-reinforced epoxy resin core rod and end fittings [9]. Due to the Si–O bond of silicone rubber and its inorganic properties, silicone rubber is superior to ordinary organic rubbers in terms of heat resistance, chemical stability, electrical insulating, abrasion resistance and weatherability [10]. The excellent hydrophobicity and hydrophobic recovery properties of silicone rubber are key factors in its use as a high-voltage outdoor insulation material. As an insulating material used outdoors for a long time, the silicone rubber umbrella skirt will gradually age under the long-term influence of humidity, surface discharge, ultraviolet rays, temperature, smoke and other factors [11,12]. The aging of the material will make the surface of the insulator hydrophobicity deteriorate, resulting in the occurrence of a leakage current and flashover phenomenon, meaning that the hydrophobicity is an important index used to examine the performance of the insulator [13,14].

To enhance the hydrophobicity of silicone rubber, researchers have used various approaches, including surface modifications, such as plasma jet treatment [15,16], spraying [17], laser irradiation [18], or adding fillers to build up nanostructures. Mendoza et al. [19] conducted a comparative assessment of hydrophilic and hydrophobic ZnO nanoparticles and their methods of deposition on the surface hydrophobicity of silicone rubber (PDMS) and glass substrates. An accurate method was proposed to determine the contact angle hysteresis. Nazir et al. [20] added milled glass fires and graphene nanoplatelets as fillers in silicone rubber and found that the composites have excellent fire retardancy and better mechanical strength and hydrophobicity while retaining the required electrical breakdown strength. Zhu [21] believes that the mechanism through which corona discharge weakens the hydrophobicity of silicone rubber is the particles generated by the discharge continuously impacting the SR surface, on which the hydrophilic hydroxyl group is replaced by a polar hydrophobic methyl group. Khan et al. [22] prepared room temperature vulcanized silicone rubber composites, and after 9000-hour aging tests, it was found that the samples using silica as fillers had better hydrophobicity, and the samples with aluminum trihydrate as filler had higher dielectric breakdown strengths. Sheng et al. [23] added a glycerol layer onto the surface of the silicone rubber, and the contact angle of the silicone rubber could be improved by 19.9% via irradiation treatment with glycerol. Du et al. [24] fluoridated the silicone rubber using fluorine gas to obtain the sample with a contact angle of 116°. Surface modification often requires high costs and is a complicated procedure. And these material improvement methods can make silicone rubber significantly improved in a certain aspect, but do not comprehensively consider the purpose of using it at low temperatures. Therefore, it is recommended to use a fluorosilicone rubber (FSIR) and methyl vinyl silicone rubber (SIR) blend to take into account the three aspects of hydrophobicity, insulation and low-temperature resistance.

FSIR has methyl, vinyl and trifluoropropyl side chains, which improve the oil and solvent resistance of the rubber due to the electronic effect and the good shielding effect of the C-F bond on the C=C bond [25,26]. It also has a wide operating temperature range of −60–200 °C and can be operated in cold environments [27]. Sun et al. [28] found that after ultraviolet aging for 2000 h, the FSIR insulators had a larger contact angle. Wei et al. [29] found that the resistance and breakdown properties of phenyl silicone rubber (SiR) were better than those of vinyl SiR and fluoro-SiR, and fluoro-SiR has a higher dielectric constant than the vinyl SiR and phenyl SiR. Polymer blending allows composites to combine the characteristics of both materials. Metivier et al. [30] found that silicone/fluorosilicon mixtures are compatible by adding surface hydrophilic silica particles, and fumed hydrophilic silica can reduce the size of the fluorosilicon phase to 500 nm. Khanra et al. [31] added modified silica in different ratios to fluoroelastomer and silicone rubber blends, which exhibited good compatibility and improved mechanical properties.

The good hydrophobicity and low-temperature resistance of FSIR are important factors conducive to the operation of insulators in harsh environments. Considering the many advantages of FSIR, in this work, FSIR and SIR were used to prepare composites with different ratios. The expectation is that the SIR/FSIR composites can be used in high-voltage insulators in a harsh environment. Scanning electron microscopy (SEM) tests, tensile tests, dynamic mechanical property tests and contact angle tests were carried out to analyze the effects of different ratios on the micro-morphology, mechanical properties, crosslinking density, crystallization temperature and hydrophobicity of the composites. It is proved that the addition of FSIR can improve the material’s hydrophobicity and enhance its low-temperature resistance.

## 2. Experimental

### 2.1. Materials

The vinyl content of SIR (XHG-110) was 0.08%, and its molecular weight was 670,000; the material was produced by Zhejiang Wynca Chemical Group Co., Jiande, Zhejiang, China. The vinyl content of FSIR (MFVQ 1402) was 0.35%, and its molecular weight was 730,000; the material was produced by Shenzhen Oufut Rubber Products Co., Ltd., Shenzhen, Guangdong, China. Silica adopted HDK^®^V15 from Wacker, Germany, and its density was 2.2 g·cm^−3^, its specific surface area was 130–170 m^2^·g^−1^, and its purity was 99.8%. Hydroxy silicone oil (HSO), aluminum hydroxide (ATH), vinyltrimethoxysilane (VTMS), ferric oxide (Fe_2_O_3_), 2,5-dimethyl-2,5-di(tert-butylperoxy) hexane (DBPH) and other reagents were commercially available. Table 1 shows the experimental formulations of the composites.

### 2.2. Preparation

Firstly, SIR was mixed with the reagents of reinforcing agent silica, thermal conductivity enhanced agent ATH, silane coupling agent VTMS, structural control agent HSO, coloring agent Fe_2_O_3_ and vulcanizing agent DBPH in an open two-roll mill. The fillers were added in sequence, and the SIR was taken out after uniform mixing. Secondly, FSIR was prepared following the same procedure. Then, SIR and FSIR were mixed in different ratios in the opening machine to obtain different ratios of SIR/FSIR compounds. Finally, after the corresponding optimum curing time(t_90_) was measured using a vulcanizing instrument (MDR-2000, Shanghai Dejie Machine Equipment Co., Ltd., Shanghai, China), vulcanization was carried out using a flatbed vulcanizing machine (XLB-0350, Zhejiang Huzhou Dongfang Machinery Co., Ltd., Huzhou, Zhejiang, China) at 170 °C.

### 2.3. Characterization and Measurements

A cold field emission scanning electron microscope (SU8020, Hitachi, Tokyo, Japan) was used to observe the tensile fracture surface of the samples.

The mechanical properties of the composites were tested using a universal material tester (GT-TC2000, Gotech Testing Machines Inc., Taizhong, Taiwan, China), which had a tensile speed of 500 mm·min^−1^.

The crosslinking density of the composites was tested via the equilibrium swelling method [32]. The mass of the samples before and after immersion were defined as *M*_1_ and *M*_2_ (g), which were obtained by immersing the vulcanized rubber in toluene at room temperature for 72 h. The volume rate of swelling of the rubber, *v*_2_, was calculated according to Equations (1)–(3).

(1) $$\nu_{2}=\frac{\nu_{1}}{\left(\nu_{1}+\nu_{sol}\right)}$$

(2) $$\nu_{sol}=\frac{\left(M_{2}-M_{1}\right)}{\rho_{sol}}$$

(3) $$\nu_{1}=\frac{M_{3}}{\rho }$$

where *v_sol_* (cm^3^·mol^−1^) is the volume of solvent absorbed by the rubber after swelling, *v*_1_ (cm^3^·mol^−1^) is the volume of rubber, *ρ_sol_* (g·mL^−1^) is the density of solvent, *ρ* (g·cm^−3^) is the density of rubber and *M*_3_ (g) is the mass of rubber. The crosslinking density, *v_e_* (×10^−4^ mol·cm^−3^), of the composites was calculated using Equation (4).

(4) $$\nu_{e}=\frac{\ln\left(1-\nu_{e}\right)+\nu_{2}+\chi \cdot \nu_{2}^{2}}{2\nu \cdot \nu_{2}^{\frac{1}{3}}}$$

where *χ* is the silicone rubber–toluene interaction parameter (0.465) [33], and *v* (cm^3^·mol^−1^) is the molar volume of solvent.

The dynamic mechanical analysis (DMA) of the silicone rubber composites were tested using DMA 242 (NETZSCH, Free State of Bavaria, Germany). The tests were carried out at a frequency of 10 Hz, an amplitude of 0.5% and temperature conditions of −180 to 25 °C.

The breakdown strength tests used a voltage breakdown tester manufactured by Beijing Huaji Instrument Co., Beijing, China. The test was performed at an AC voltage of 50 Hz. The insulating properties of the composites were analyzed using the Weibull classical failure model [34]. Equation (5) represents the failure distribution function.

(5) $$F\left(x\right)=1-e^{-{\left(\frac{x}{\alpha }\right)}^{\beta }}$$

where *x* is the breakdown strength, *α* is the scale parameter, and *β* is the shape parameter. To facilitate the calculation, the above formula can be converted to logarithmic form, as shown in Equation (6).

(6) lg[−ln(1−F)]=β(lgα−lgx)

In addition, the failure distribution function can be calculated using Equation (7).

(7) $$F\left(x\right)=\frac{i-0.5}{n+0.25}$$

where *i* is the number of measurements derived by arranging *x* in ascending order, and *n* is the total number of tests for each sample—in this work, *n* = 12. The relevant parameters *α* and *β* of the Weibull distribution function were calculated using the least squares method for Equation (6) to derive the Weibull failure model.

The static contact angle (SCA) and hydrophobic migration of the composites were measured using a contact angle meter manufactured by Shanghai Zhongchen Technical Equipment Co., Shanghai, China. Before the test took place, the samples were sequentially wiped with ethanol and ultrapure water and left for 24 h. The SCA test was carried out after the natural evaporation of the water on the surface. A droplet of water with a volume of about 20 μL was dropped on the surface of the sample using a microsyringe. After taking pictures to record the shape of the droplet, five points on the boundary were selected, and the coordinates were recorded. SCA was obtained via fitting. The final result was the average value of five measurements that were taken for each group. For the hydrophobic migration test, the samples were immersed in ultrapure water for 96 h, after which stage the SCA test was performed.

## 3. Results and Discussion

### 3.1. Mechanical Properties

**Figure 1:**
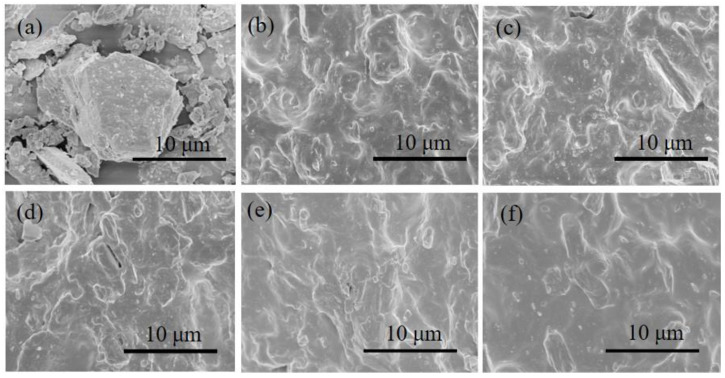
SEM microstructure of tensile sections of the SIR/FSIR composites: (**a**) ATH; (**b**) 100/0; (**c**) 90/10; (**d**) 80/20; (**e**) 70/30; (**f**) 0/100.

SEM testing can be used to observe the dispersion of fillers in the matrix. To observe the microscopic topography of the prepared sample, the cross-section after fracture in the tensile test was observed, and the compatibility of the fillers with rubber was analyzed to carry out the blending scheme. Figure 1a shows micron-sized ATH, which can be seen to have a layered structure. ATH has good adsorption and dispersion, which can improve the thermal conductivity of rubber, enhance the anti-aging effect and strengthen the vulcanization process. However, the addition of FSIR will affect the compatibility of ATH and the rubber matrix, and the tensile section of the sample was analyzed via SEM. Figure 1b shows the SIR composite. The ATH, which is exposed in the outer layer of the silicone rubber matrix, cannot be observed. Figure 1f shows the fracture in the FSIR composites. The section is smoother and flatter than that of the SIR composites, indicating that its compatibility is better. In Figure 1c–e, unwrapped ATH and tiny cracks can be observed in SEM. Significant bulges and depressions due to material agglomeration can be observed compared to pure FSIR or pure SIR composites. The scheme in which SIR/FSIR is 70/30 shows better compatibility, and the reason for this phenomenon is that the increase in the FSIR content promotes the fusion of ATH and rubber. Therefore, for SIR/FSIR, the interfacial interaction between the matrix and the filler is weaker, resulting in the mechanical properties of the materials being lower than those of the SIR composite materials.

**Figure 2:**
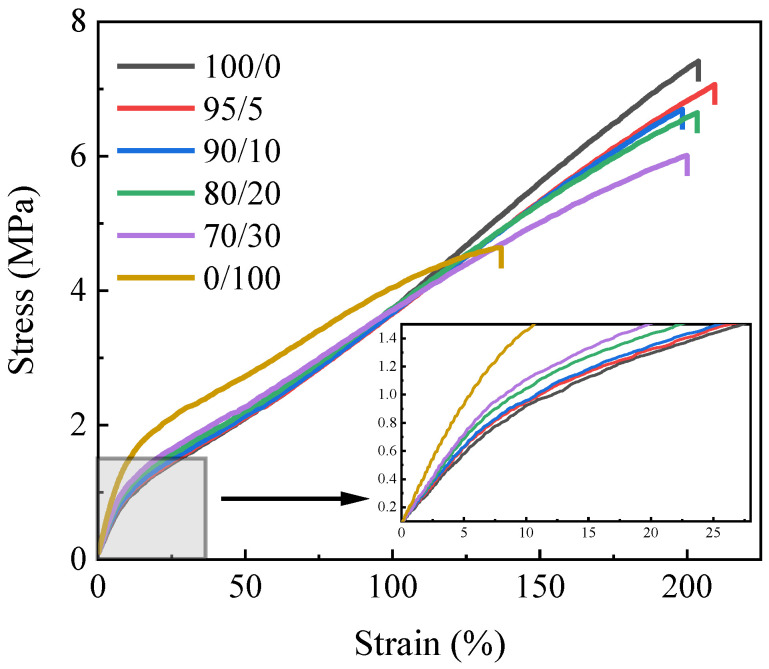
Stress–strain curves of SIR/FSIR composites.

Figure 2 shows the stress–strain curves of the SIR/FSIR composites. The elongation at break undergoes very little change with the increase in FSIR contents, which were all around 205%. Table 2 lists the mechanical properties of the SIR/FSIR composites. The tensile strength of the SIR composite is up to 7.3 MPa, while the tensile strength decreases to 5.9 MPa when the content of FSIR is increased to 30 phr. In JB/T 10945-2010, the silicone rubber applied to composite insulators requires the tensile strength to be higher than 3 MPa. Therefore, even if the tensile strengths of the blend composites are reduced via the addition of FSIR, it still meets the industry standard for silicone rubber insulators. The elongation at break of the FSIR composite is only 138%, and the tensile strength is 4.5 MPa, which are much lower than those of the SIR/FSIR composites. When the stress is less than 1.5 MPa, the modulus of elasticity of the composites tends to increase with the increase in the FSIR content. This outcome occurs due to the fact that the FSIR side chain contains a small amount of trifluoropropyl, and the presence of fluorine atoms makes the molecule more polar. This process results in larger intermolecular forces and reduced molecular chain motility. In addition, for SIR/FSIR composites, the hardness of the composites increases, and the stress at 100% strain remains stable, which is about 3.6 MPa with the increase in the FSIR content. Since the percentage of SIR is higher than that of FSIR in the four composites analyzed in this work, the properties of the samples are more similar to those of SIR.

### 3.2. Crosslinking Density

**Figure 3:**
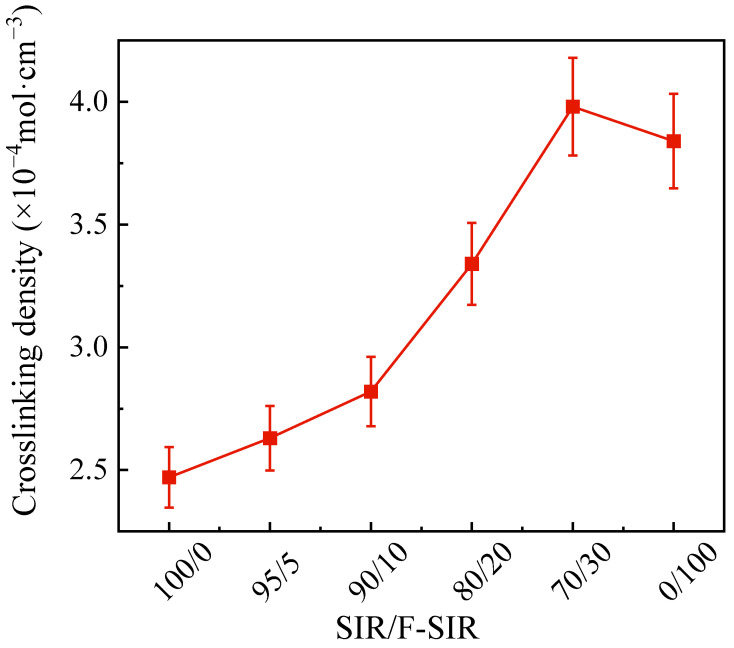
Crosslinking density of SIR/FSIR composites.

After the blending rubber is vulcanized, a cross-linked network is formed inside of the structure. When the crosslinking density increases, it represents the weakening of the motility of the macromolecular chain. Therefore, this test can provide a theoretical analysis from a microscopic perspective for tensile testing. The crosslinking density of the SIR/FSIR composites is given in Figure 3. It is found that the crosslinking density of FSIR composite is 1.55 times that of SIR composite. For the SIR/FSIR composites, the crosslinking density gradually increases with the increase in FSIR content. When the proportion of FSIR increased from 5 to 20 phr, the crosslinking density increased from 2.63 × 10^−4^ to 3.34 × 10^−4^ mol·cm^−3^. The increased crosslinking density indicates that the connection between the macromolecular chains is more compact, and the flexibility of the composite material is reduced and the elastic modulus will increase. In Table 1, the elasticity modulus of FSIR composite is 7.8 MPa higher than that of SIR composites. The macromolecular chain of FSIR contains a small amount of trifluoropropyl, and the electron-absorbing effect of fluorine atoms is stronger and, thus, the free radical reactions are more likely to occur with the vulcanizing agent. As a result, compared to the SIR composite, the SIR/FSIR composites with higher FSIR contents show higher crosslinking densities. However, the crosslinking density of the scheme with an SIR/FSIR ratio of 70/30 is higher than that of the pure FSIR composites. The reason for this outcome is that silica is more likely to interact with non-polar molecules to form crosslinking networks. Therefore, there is an optimal value between the proportions of SIR composites and FSIR composites that maximizes the crosslinking density.

### 3.3. Dynamic Mechanical Properties

**Figure 4:**
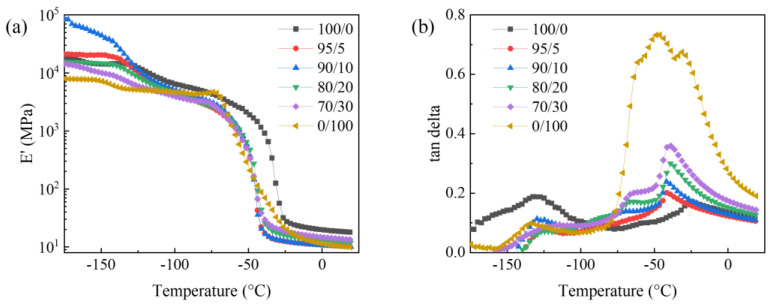
DMA of SIR/FSIR composites: (**a**) storage modulus–temperature curve; (**b**) tan delta–temperature curve.

Figure 4a shows the storage modulus–temperature curve of the SIR/FSIR composites. The storage modulus of pure SIR is higher than that of pure FSIR under all of the test temperatures. Figure 4b shows the tan delta–temperature curve of SIR/FSIR composites obtained from DMA. The glass-transition temperatures are about −130 °C for all of the blending schemes. When the SIR/FSIR composites are in a glassy state, the storage modulus of the composites gradually decreases as the proportion of FSIR increases.

It can be seen that there are two damping peaks in FSIR, in which the temperature of the crystallization peak is about −50 °C, as shown in Figure 4b. The temperature of the crystallization peak of SIR is about −30 °C. Therefore, FSIR has a superior low-temperature resistance. When SIR/FSIR is 95/5, there is only one crystallization peak, indicating that the compatibility of the two matrices is better at this time. When SIR/FSIR is 70/30, two crystalline peaks are observed, which means that two crystalline phases have been included in the composites. The crystallization peak of FSIR is obviously larger than that of SIR, which occurs because the increase in trifluoropropyl makes the internal polarity of the material larger. The reduction in the crystallization temperature can extend the operating temperature range of the composite. The crystallization temperatures of the larger peaks are concentrated at around −45 °C, indicating that the low-temperature resistance of the composites is significantly improved by the addition of FSIR.

### 3.4. Breakdown Strength

**Figure 5:**
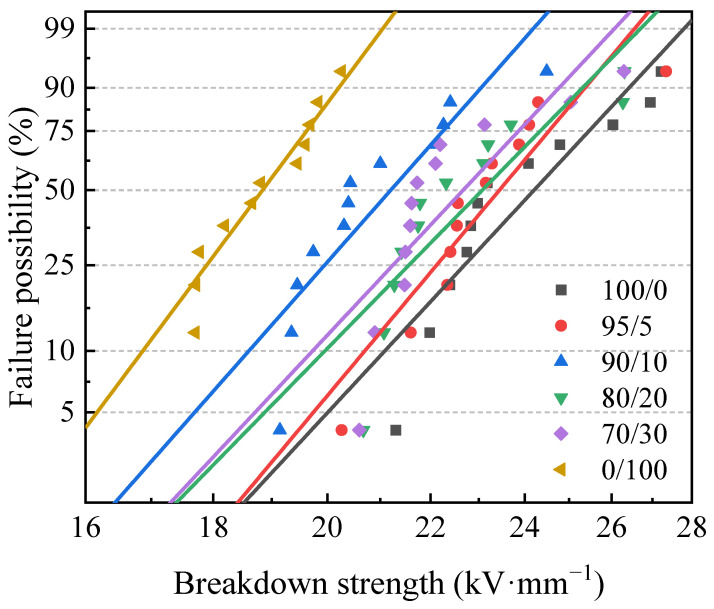
Weibull distributions of SIR/FSIR composites.

As an insulator umbrella sleeve material, its insulation performance is one of the most important indicators. The breakdown strength of the composites was investigated, and the Weibull distribution was obtained. Figure 5 shows the Weibull distribution of SIR/FSIR composites. It can be seen that the SIR composite material has excellent insulation properties. The presence of trifluoropropyl makes the molecule polar, which makes the breakdown field strength of the sample smaller and easier to breakdown. SIR/FSIR composites contain two crystalline phases, resulting in more structural defects and reduced insulation properties. Therefore, based on the characterization data, it can also be confirmed that the breakdown strength of the material decreases as the proportion of FSIR in the composites increases. When the content of FSIR is lower, the compatibility between SIR and FSIR is greater, and the decrease in breakdown strength is smaller. Therefore, for the SIR/FSIR composite with 5 phr FSIR, the breakdown strength is comparable to that of SIR composite. However, as the blending ratio of FSIR continues to increase, the breakdown strength continues to decrease. It can be seen that adding excess FSIR will lead to a decrease in the insulation performance of the SIR/FSIR composite. 

### 3.5. Static Contact Angle

**Figure 6:**
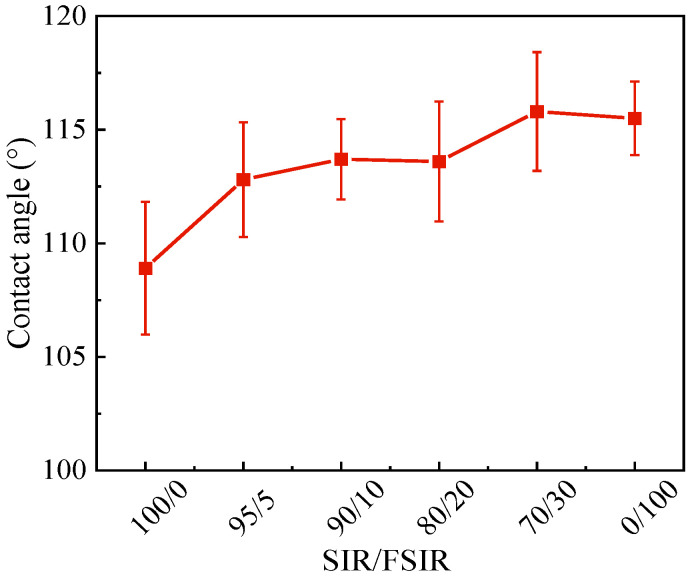
Static contact angles of SIR/FSIR composites.

The hydrophobicity of composites is characterized by contact angle testing. When the SCA is larger, the sample surface has better hydrophobicity. Sun et al. [28] tested the contact angle of the two insulator materials of SIR composites and FSIR composites, which were 112.4° and 117.8°. Figure 6 shows the SCA of SIR/FSIR composites. The SCAs are 108.9° and 115.5° for SIR composite and FSIR composite, respectively. The difference in the hydrophobicity of this paper and the work of Sun may be due to experimental formulations and test methods. FSIR has a lower surface free energy, meaning that its hydrophobicity is stronger than that of SIR. In addition, for the SIR/SIR composites, the SCA gradually increases with the increase in the FSIR content. This finding indicates that the higher the content of FSIR, the greater the hydrophobicity of the composites. When the proportion of FSIR is increased to 5 phr, the SCA can be significantly increased by 3.9°. With the SIR/FSIR increasing to 90/10 and 80/20, the SCA is about 113.6° and shows a smooth trend. For the scheme of 70/30, the SCA is slightly higher than the FSIR composites. This observation is the same as the change law of crosslinking density, meaning that the intertwining of molecular chains inside of the material to form a dense crosslinking network is conducive to improving hydrophobicity.

**Figure 7:**
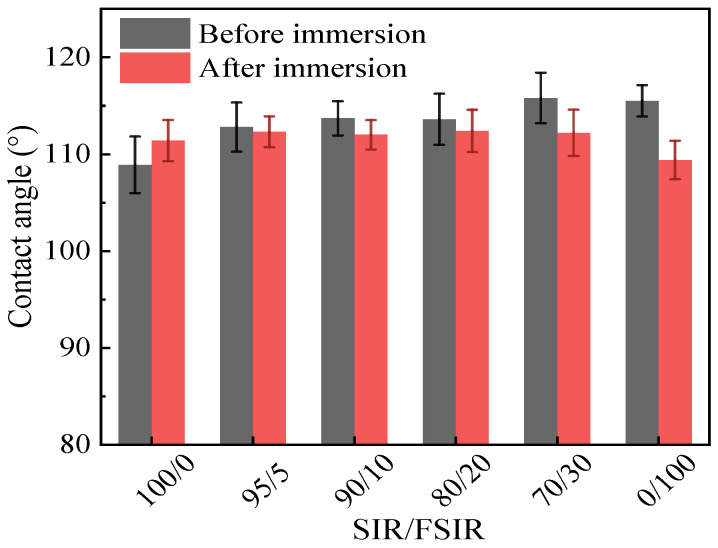
Migration of hydrophobicity in SIR/FSIR composites.

When the sample is served in a humid environment for a long time, its hydrophobicity changes. Therefore, hydrophobic migration was analyzed. Figure 7 shows the variation in SCA for SIR/FSIR composites measured after 96 h of immersion in water. After 96 h of immersion, the SCA of FSIR composite decreases more significantly than that of SIR composite. For the SIR/FSIR composites, the decreasing trend of SCA is more significant for the increased proportion of FSIR. When SIR/FSIR is 95/5, the SCA decreases by 0.5° after 96 h immersion, while the SCA decreases by 3.6° when SIR/FSIR is 70/30. This phenomenon indicates that the FSIR material has good surface hydrophobicity, but the hydrophobicity migration occurred after internal water immersion. In addition, the SCA of the blended material with FSIR composites after immersion is higher than that of the un-immersed SIR composites. For example, the smallest SCA measured after immersion of all SIR/FSIR blending composites was 112° for the scheme 90/10, but it is still larger than the un-immersed SIR composite. Based on this result, we can further illustrate the effectiveness of FSIR composite in improving hydrophobicity. Insulators made of SIR/FSIR can have better hydrophobic stability in humid environments.

## 4. Conclusions

In this paper, SIR/FSIR composites with different proportions of matrix materials were prepared and found to have good compatibility. Firstly, the tensile strength decreased from 7.3 to 5.9 MPa when the content of FSIR was increased to 30 phr. The elongation at break and the stress at 100% strain did not significantly change. When the stress was less than 1.5 MPa, the modulus of elasticity of the composites tended to increase with the increase in FSIR content. Secondly, the low-temperature resistance of the composites can be significantly improved when the proportion of FSIR in the composites is relatively small. Compared to SIR composite materials, the crystallization temperature of the composites is reduced from −30 to −45 °C. Thirdly, the breakdown strength test reveals that when the FSIR content is increased, it leads to a decrease in the overall insulating properties of the composites. Finally, the SCA increases as the FSIR content increases for blending composites, and the material hydrophobicity increases. When the samples were immersed for 96 h, the hydrophobicity migration phenomenon occurred. As the proportion of FSIR increased, the SCA decreased more significantly, which indicated that hydrophobicity weakened.

This research provides a reference point for the material formulation of SIR high-voltage insulators. The huge demand for composite insulators has been shown in power transportation, especially in cold weather or harsh environments. Insulators composed of SIR/FSIR composites exhibit the advantage of having wider temperature application range. In addition, owing to the increased hydrophobicity, the insulators using SIR/FSIR composites as an umbrella sleeve material are expected to efficiently avoid flashover, which could prolong the service life of the insulator and reduce the probability of the need to replace the disabled insulator.

## Figures and Tables

**Table 1:** Experimental formulations of SIR/FSIR composites (phr).

Ingredients	100/0	95/5	90/10	80/20	70/30	0/100
SIR	100	95	90	80	70	0
FSIR	0	5	10	20	30	100
Silica	30	30	30	30	30	30
ATH	100	100	100	100	100	100
Fe_2_O_3_	4	4	4	4	4	4
HSO	5	5	5	5	5	5
VTMS	2	2	2	2	2	2
DBPH	0.5	0.5	0.5	0.5	0.5	0.5

**Table 2:** Mechanical properties of SIR/FSIR composites.

Properties	100/0	95/5	90/10	80/20	70/30	0/100
Shore A hardness	70	71	70	72	74	77
Tensile strength (MPa)	7.3	6.9	6.6	6.5	5.9	4.5
Elongation at break (%)	204	210	199	204	200	138
Stress at 100% strain (MPa)	3.7	3.6	3.5	3.7	3.6	4.0
Elasticity modulus (MPa)	13.3	14.1	14.1	15.0	16.3	21.1

## Data Availability

Not applicable.

## Nomenclature

SIR	Silicone rubber
FSIR	Fluorosilicone rubber
HSO	Hydroxy silicone oil
ATH	Aluminum hydroxide
VTMS	Vinyltrimethoxysilane
DBPH	2,5-dimethyl-2,5-di (tert-butylperoxy) hexane
SEM	Scanning electron microscope
DMA	Dynamic mechanical properties
SCA	Static contact angle
